# Myeloperoxydase and CD15 With Glycophorin C Double Staining in the Evaluation of Skin Wound Vitality in Forensic Practice

**DOI:** 10.3389/fmed.2022.910093

**Published:** 2022-05-17

**Authors:** Guillaume Gauchotte, Agathe Bochnakian, Philippe Campoli, Emilie Lardenois, Muriel Brix, Etienne Simon, Sophie Colomb, Laurent Martrille, Pierre-Antoine Peyron

**Affiliations:** ^1^Department of Biopathology, CHRU-ICL, CHRU Nancy, Vandoeuvre-lès-Nancy, France; ^2^Faculty of Medicine, Université de Lorraine, Vandoeuvre-lès-Nancy, France; ^3^Department of Legal Medicine, CHRU Nancy, Vandoeuvre-lès-Nancy, France; ^4^INSERM U1256, NGERE, Vandoeuvre-lès-Nancy, France; ^5^Centre de Ressources Biologiques, BB-0033-00035, CHRU, Nancy, France; ^6^Department of Maxillofacial and Plastic Surgery, CHRU, Nancy, France; ^7^Department of Forensic Medicine, CHU Montpellier, University of Montpellier, Montpellier, France; ^8^EDPFM, University of Montpellier, Département de Médecine Légale, Montpellier, France; ^9^IRMB, INM, University of Montpellier, INSERM, CHU Montpellier (LBPC-PPC), Montpellier, France

**Keywords:** CD15, myeloperoxidase (MPO), glycophorin, vitality, wound datation, histology, immunohistochemistry, forensic

## Abstract

**Background:**

The determination of skin wound vitality based on tissue sections is a challenge for the forensic pathologist. Histology is still the gold standard, despite its low sensitivity. Immunohistochemistry could allow to obtain a higher sensitivity. Upon the candidate markers, CD15 and myeloperoxidase (MPO) may allow to early detect polymorphonuclear neutrophils (PMN). The aim of this study was to evaluate the sensitivity and the specificity of CD15 and MPO, with glycophorin C co-staining, compared to standard histology, in a series of medicolegal autopsies, and in a human model of recent wounds.

**Methods:**

Twenty-four deceased individuals with at least one recent open skin wound were included. For each corpse, a post-mortem wound was performed in an uninjured skin area. At autopsy, a skin sample from the margins of each wound and skin controls were collected (*n* = 72). Additionally, the cutaneous surgical margins of abdominoplasty specimens were sampled as a model of early intravital stab wound injury (scalpel blade), associated with post-devascularization wounds (*n* = 39). MPO/glycophorin C and CD15/glycophorin C immunohistochemical double staining was performed. The number of MPO and CD15 positive cells per 10 high power fields (HPF) was evaluated, excluding glycophorin C—positive areas.

**Results:**

With a threshold of at least 4 PMN/10 high power fields, the sensitivity and specificity of the PMN count for the diagnostic of vitality were 16 and 100%, respectively. With MPO/glycophorin C as well as CD15/glycophorin C IHC, the number of positive cells was significantly higher in vital than in non-vital wounds (*p* < 0.001). With a threshold of at least 4 positive cells/10 HPF, the sensitivity and specificity of CD15 immunohistochemistry were 53 and 100%, respectively; with the same threshold, MPO sensitivity and specificity were 28 and 95%.

**Conclusion:**

We showed that combined MPO or CD15/glycophorin C double staining is an interesting and original method to detect early vital reaction. CD15 allowed to obtain a higher, albeit still limited, sensitivity, with a high specificity. Confirmation studies in independent and larger cohorts are still needed to confirm its accuracy in forensic pathology.

## Introduction

The determination of skin wound vitality is a challenge for the forensic pathologist. The detection of an inflammatory infiltration based on histology is to date the gold standard, being highly specific but showing a very low sensitivity in recent wounds. Most notably, in the first minutes or hours after the infliction of a wound, standard histological examination may not determine whether the wound was inflicted in pre- or post-mortem period. Indeed, the delay before the detection of first polymorphonuclear neutrophils (PMN) infiltration may vary from 10 min to 6 h (1, 2). Immunohistochemistry (IHC) is a cost effective and easy-to-use method that could allow to obtain a higher sensitivity (3). Upon the candidate markers, CD15 and myeloperoxidase (MPO) may allow to early detect PMN (4–7). However, one potential pitfall on IHC slide is the difficulty to differentiate passive extravasation of PMN in hemorrhagic infiltration from true active diapedesis. The association with a red blood cells marker, like glycophorin C, could allow to increase specificity, by avoiding the count of inflammatory cells in hemorrhagic areas.

The aim of this study was to evaluate the sensitivity and the specificity of CD15 and MPO, with glycophorin C co-staining, in comparison with standard histology, in a series of recent medicolegal wounds and post-mortem controls, and in a prospective human experimental surgical model.

## Materials and Methods

### Study Population and Sample Collection

#### Medicolegal Wounds

Twenty-four individuals (20 men, 4 women, mean age = 51.0 ± 24.3 years) with at least one recent open skin wound were included at the mortuary of the University Hospital of Montpellier. Skin wounds consisted in 20 lacerations from polytrauma cases (traffic accidents, falls from high height) and 4 gunshot wounds. They were mostly located on the lower limbs and the torso. The time interval between trauma and death (survival time) was determined with medical records and police reports, including testimony from witnesses. It varied from a few seconds to 180 min (mean = 47 min). Bodies displaying putrefactive changes were excluded from the study, as well as individuals with severe malnutrition, known immunodeficiencies and immunotherapy.

For each corpse, a post-mortem 2 cm-incision was performed with a scalpel in an uninjured skin area contralateral to the ante-mortem wound, shortly after arrival at the mortuary and before refrigeration. The elapsed time between death and the infliction of the post-mortem wound was comprised between 0 and 180 min (median: 40 min). In patients with multiple ante-mortem wounds, the wound of interest was selected based on its location (no skin sample was collected from the head or hands, for ethical reasons) and on its size (large wounds were preferred to small ones).

At autopsy, a skin sample from the margins of each wound (ante- and post-mortem) and from an uninjured skin area located on the midline incision line (control samples) were collected on each corpse and immediately placed for fixation in 10% buffered formalin solution. A total of 72 skin samples were removed, including 24 samples from each of the conditions: ante-mortem wounds, post-mortem wounds, and healthy skin (control samples). The average post-mortem interval at the time of sampling was 66.3 ± 28.3 h (24–117 h).

#### Surgical Wounds

As a model of recent vital stab wound injury, the cutaneous surgical margins of abdominoplasty specimens were prospectively collected at the Department of Maxillofacial and Plastic Surgery of the University Hospital of Nancy, France. The precise time interval between incision and devascularization was recorded for each margin, ranging from 0 to 61 min (median: 24 min). As a model of early post-mortem wounding, a wound was inflicted with a sterile scalpel in the center of the specimens, 5 min. after devascularization. In the Pathology Department, tissue sampling was performed perpendicularly to the skin margins, on fresh tissue, before fixation in buffered formalin solution. Thirty-nine samples (26 pre-devascularization and 13 post-devascularization) were obtained from 13 patients.

### Standard Histology

After formalin fixation and paraffin embedding, 5-μm sections were stained with hematoxylin, eosin, and saffron (HES), and a blind histological examination of the vital and post-mortem wounds was performed, taking into account the presence or absence of hemorrhagic infiltration and counting the number of PMN in 10 consecutive high-power fields (HPF) (×400 magnification; 0.237 mm^2^).

### Immunohistochemistry

Paraffin 5-μm sections were immersed in a 10 mM sodium citrate buffer (pH6) for 20 min at 97°C for dewaxing and antigen retrieval. The following primary antibodies were used (30 min incubation): CD15 (mouse monoclonal, ready-to-use, DakoCytomation Agilent, Glostrup, Denmark); myeloperoxidase (MPO) (rabbit polyclonal, ready-to-use, Dako); glycophorin C (mouse monoclonal, 1/40, Diagnostic BioSystems). Immunohistochemistry was performed with Dako Autostainer Plus (Dako) using Flex Envision revelation system (Dako) for CD15 or MPO, followed by Envision Magenta (Dako) for glycophorin C. Appropriate positive and negative controls were used throughout the experiment.

A quantitative evaluation of staining for CD15 and MPO was counted in 10 consecutive HPF (0.237 m^2^/field) on one representative slide per case, in the immediate vicinity of the wound margin, from the superficial dermis to the deep subcutaneous adipose tissue, taking into account all interstitial leucocytes showing stained cytoplasm, excluding intravascular cells and those within hemorrhagic areas, these latter being underlined by the anti-glycophorin C antibody.

### Statistical Analyses

The presence of absence of interstitial hemorrhage was considered as a qualitative variable, and the numbers of PMN, positive MPO and positive CD15 cells as quantitative variables. Statistical analysis was performed with IBM SPSS Statistics version 27.0. software. To compare the different groups (ante-mortem vs. post-mortem; pre-devascularization vs. post-devascularization), the Fisher exact test was used for qualitative variables and the Mann-Whitney Wilcoxon test for quantitative variables. The correlation between quantitative variables was evaluated with the Spearman’s coefficient correlation. A *p*-value lesser than 0.05 was considered as statistically significant.

Ante-mortem and pre-devascularization wounds were considered as vital wounds, whereas post-mortem wounds, control samples, and post-devascularization wounds were defined as non-vital wounds. For the evaluation of sensitivity and specificity, true positivity was defined by a cell count number equal or greater to the defined threshold in vital skin wounds; false positivity by a number equal or greater to the defined threshold in non-vital samples; true negativity by a number lesser to the threshold in non-vital samples; false negativity by a number lesser to the threshold in vital wounds. Receiving operating characteristic (ROC) curves were performed for each marker, in order to screen for the most optimal threshold.

## Results

### Evaluation of Inflammation With Standard Histology

**FIGURE 1:**
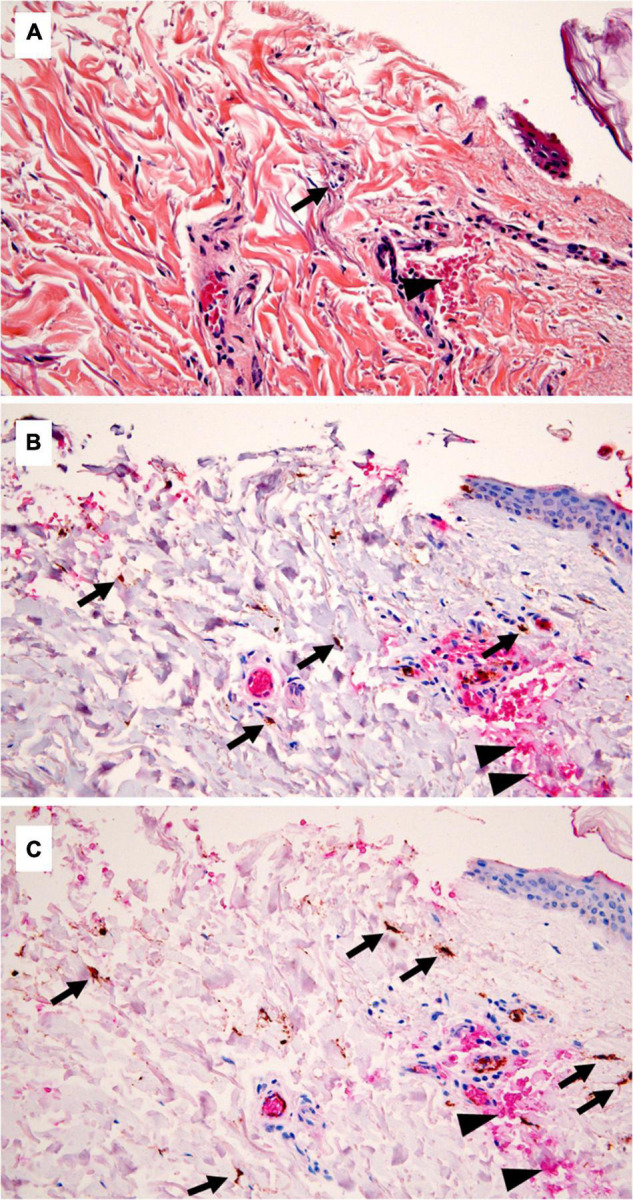
Standard histology and immunohistochemistry (IHC) in a medicolegal wound. **(A)** Standard histology showing only one polymorphonuclear (PMN) (arrow) close to the wound margin, and hemorrhagic infiltration (arrowhead) (hematoxylin, eosin, and saffron, × 400 magnification). **(B)** Double staining for myeloperoxidase (MPO) and glycophorin C, underlining the presence of several inflammatory cells (arrows), evaluated outside the hemorrhagic areas (glycophorin C—positive, arrowheads) to avoid the count of passively extravasated leucocytes (IHC, ×400). **(C)** CD15/glycophorin C double staining, showing a greater number of positive cells, comparing with MPO (IHC, ×400).

In the medicolegal wounds, no significant inflammatory reaction was seen on standard histology slides (Figure 1A). In the surgical wounds, a significant inflammatory reaction was found in 2 cases, showing a PMN infiltration (7 and 31 PMN/10 HPF). PMN evaluation showed a median number of 1 PMN/10 HPF (min.-max.: 0–31) in vital wounds and 1 PMN/10 HPF (min.-max.: 0–3) in non-vital wounds (Table 1). No significant difference was found between vital and non-vital wounds, in both autopsy cases and surgical wounds (*p* = 0.557 and *p* = 0.294, respectively).

**TABLE 1:** Evaluation of the median number of polymorphonuclears neutrophils (PMN) on standard histology and results of anti- myeloperoxydase (MPO) and CD15 immunohistochemistry, in the autopsy cohort and in the surgical model [median (min.-max.); * statistically significant, *p* < 0.05; Mann-Whitney Wilcoxon test].

Samples	Vital wounds	Non-vital	*p-*value	Total
**PMN**				
Autopsy	1 [0–3]	0 [0–2]	0.557	1 [0–3]
Surgery	1 [0–31]	0 [0–3]	0.294	1 [0–31]
Total	1 [0–31]	0 [0–3]	0.106	1 [0–31]
**MPO**				
Autopsy	1 [0–12]	0 [0–7]	0.015*	1 [0–12]
Surgery	2 [0–20]	0 [0–3]	0.014*	2 [0–20]
Total	2 [0–20]	0 [0–7]	<0.001*	1.5 [0–20]
**CD15**				
Autopsy	3 [0–24]	0 [0–3]	<0.001*	1 [0–24]
Surgery	5 [0–24]	1 [0–3]	0.004*	5 [0–24]
Total	4 [0–24]	0 [0–3]	<0.001*	4 [0–24]

A significant correlation between the number of PMN/10 HPF and the survival/pre-devascularization time was found in surgical wounds (rho = 0.424; *p* = 0.031), but not in autopsy wounds (rho = 0.135; *p* = 0.511).

With a threshold of at least 4 PMN/10 HPF, the sensitivity and specificity of the PMN count for the diagnostic of vitality were 16 and 100%, respectively.

### Interstitial Hemorrhage

With standard histology, a significant interstitial hemorrhage was noticed in 88% of ante-mortem wounds (Figure 1A) vs. 44% of post-mortem wounds and 17% of control skin samples. Using the anti-glycophorin C antibody, an interstitial hemorrhage was noticed in 96% of ante-mortem wounds vs. 56% of post-mortem wounds and 25% of control skin samples. In the surgical model, standard histology and anti-glycophorin C antibody showed an interstitial hemorrhage in, respectively, 88 and 96% of pre-devascularization wounds vs. 54 and 84% of post-devascularization wounds.

For the diagnosis of vitality, the sensitivity and specificity of the identification of interstitial hemorrhage on HES slides were 88 and 66%, respectively. With the anti-glycophorin C antibody, sensitivity raised to 96%, with a specificity of 50%.

### Myeloperoxidase and CD15 Immunohistochemistry

With MPO/glycophorin C IHC (Figure 1B), the number of positive cells was significantly higher in vital than in non-vital wounds [*p* < 0.001*;* median (min.-max.): 2 (0–20) vs. 0 (0–7)] (Table 1). This difference was still significant in the autopsy and surgery subgroups (*p* = 0.015 and *p* = 0.014, respectively). Similarly, with CD15/glycophorin C IHC (Figure 1C), the number of positive cells was significantly higher in vital than in non-vital wounds [*p* < 0.001; median (min.-max.): 4 (0–24) vs. 0 (0–3)]. This difference was still significant when considering the autopsy (*p* < 0.001) and surgery (*p* = 0.004) subgroups.

**FIGURE 2:**
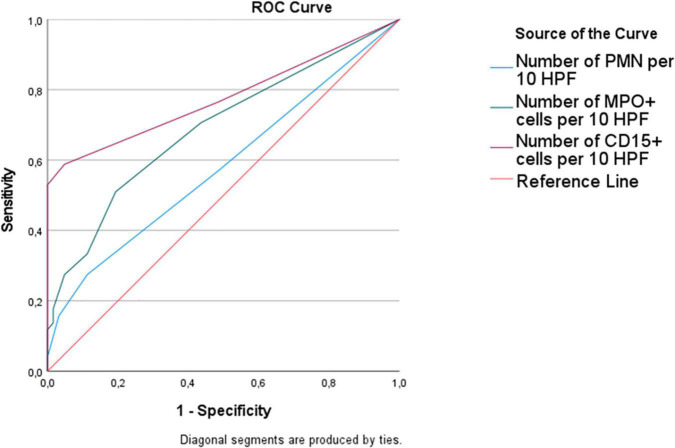
Receiver operating characteristic (ROC) curve for the diagnosis of wound vitality. The area under curve was higher for CD15 (0.78) than MPO (0.69), followed by standard histological count (0.58).

The ROC curve for the diagnosis of vitality showed that the area under the curve was higher for CD15 (0.78) than MPO (0.69) and standard PMN count (0.58) (Figure 2). With a threshold of at least 4 positive cells/10 HPF, the sensitivity and specificity of CD15 immunohistochemistry were 53 and 100%, respectively; with the same threshold, MPO sensitivity and specificity were 28 and 95%. With a threshold of at least 2 positive cells/10 HPF, the sensitivity of CD15 reached 65%, but with a lower specificity (81%). For MPO, sensitivity and specificity were 51 and 81%.

The numbers of MPO and CD15 positive cells were significantly correlated with the standard histological count for PMN (rho = 0.339, *p* < 0.001; and rho = 0.333, *p* < 0.001, respectively).

In the surgical model, the numbers of MPO and CD15 positive cells were significantly correlated with survival/pre-devascularization time (rho = 0.400, *p* = 0.043; and rho = 0.559, *p* = 0.003, respectively), but not in autopsy wounds (*p* > 0.50).

## Discussion

In the first minutes or hours, the standard histological examination may not be able to determine whether the wound was inflicted in the pre- or post-mortem period. While hemorrhagic infiltration was classically considered as a sign of vital reaction, several studies have shown that the extravasation of blood cells can also occur after death and does not represent a reliable marker in wound vitality diagnosis (8–10). Various methods can be used to detect markers of vitality, such as study of mRNAs or microRNAs (RT-PCR, *in situ* hybridization) and proteins (ELISA technique, Western blot, immunofluorescence, immunohistochemistry), focusing on the different phases of inflammation and wound healing (3). Most notably, in skin lesions, several studies about cell adhesion molecules (ICAM-1, VCAM-1, P-selectin, E-selectin, fibronectin, …) were published, reporting for some markers a good sensitivity in recent wounds, but limited by a significant risk of post-mortem false positivity (3, 11–16). More recently, the use of microRNAs in forensic science has been proposed in various applications, including wound vitality, showing for few microRNAs interesting but still preliminary results (17–19).

In this study, we propose an original method for the detection of early inflammation, based on an immunohistochemical double staining of leucocytes and red blood cells. We tested two markers of PMN: MPO and CD15. CD15 showed a higher sensitivity than MPO, which may be explained by its ability to also detect activated monocytes (20), in addition to PMN. Staining for CD15 was previously reported as a marker of early vital reaction, focusing in most of studies on brain trauma or other organs (7, 21, 22). However, as the timing and the intensity of inflammation may be influenced by the type of the trauma and the nature of the lesioned tissue (1, 23), these studies cannot be applicable to skin injuries. In skin, we previously studied this marker in surgical and medicolegal wounds (5), and it was also reported to be an interesting marker in ligature marks (6, 24) or for the assessment of the vitality in corpse dismemberment (25) and in decomposed bodies (26).

Comparing with other methods, IHC staining of inflammatory cells allows the pathologist to have a morphological control of the signal, i.e., the recognition of leucocyte shape and the precise localization within the sample. In addition to anti-CD15 or MPO antibodies, we performed a double staining with the anti-glycophorin C antibody. Glycophorin C, like glycophorin A and D, is a sialylated glycoprotein in human erythrocytes membranes. Anti-glycophorin immunohistochemistry has been proposed in various studies for the identification of the hemorrhagic infiltration, most notably in decomposed bodies or in specific conditions, such as Amussat’s sign or retinal hemorrhage (27–31). In our study, the association with the anti-glycophorin C antibody limits the risk of counting leukocytes originating from a passive extravasation of PMN in hemorrhagic infiltration, because red blood cells are more difficult to detect on IHC slides. It may also allow to detect more easily the wound margins.

The limit of this method is a relatively low sensitivity in very recent wounds, albeit higher than standard histology. The sensitivity is closely related to the type of wound or experimental model. We included in the present study only recent wounds, with a survival or pre-devascularization time of few seconds or minutes in a significant number of cases, which may explain the low sensitivity, whatever the method. In the same series of medicolegal wound, we previously found a sensitivity of 21% for the evaluation of IL8 staining, which reached 46% when using IL8 in a multiplex immunoassay, normalized on healthy skin levels (32, 33). Hence, we can conclude that IHC is less sensitive that immunoassay, but the latter has the disadvantage of needing fresh frozen tissue and to be a method which is not as largely developed as IHC, requiring training sets and data normalization, without morphological control.

Measures of test accuracy such as sensitivity and specificity depend crucially on the selected threshold, and the optimal value of this threshold is a key question for forensic practice. Given the fact that a 100% sensitivity is probably unreachable in very recent wound, we aimed to obtain a theatrical 100% specificity, to strictly avoid false positivity and obtain a high positive predictive value for vitality assessment. A threshold of 4 CD15-positive per 10 HPF cells allowed to obtain a 100% specificity and would be probably more relevant that a lower threshold, which exposes to false positivity, albeit with better sensitivity. In a previous study in which we compared CD15, FVIIIra and tryptase in medicolegal stab wounds showing inflammation and in surgical specimens (breast reductions), we found similar results for CD15, with the same threshold of 4 cells per 10 HPF (sensitivity: 47%; specificity: 100%) (5). CD15 had also the advantage to show a very good inter-observer reproducibility (0.90) (5).

## Conclusion

In conclusion, based on a series of recent medicolegal wounds associated with post-mortem controls and an experimental human model of surgical wounds, we showed that combined CD15/glycophorin C double IHC staining is an interesting and original method to detect early vital reaction. In comparison with standard histology and MPO staining, CD15 allowed to obtain a significantly higher, albeit still limited, sensitivity, with a high specificity. Confirmation studies in independent and larger cohorts are still needed in order to confirm its accuracy in forensic pathology.

## Data Availability Statement

The raw data supporting the conclusions of this article will be made available by the authors, without undue reservation.

## Ethics Statement

The post-mortem study protocol was approved by the French Agency of Biomedicine, Nr. PFS15-003. The surgical model protocol was approved by the review board of the Direction of Research and Innovation, CHRU of Nancy, France (CPRC2013, DRCI, CHRU Nancy) and a written consent was obtained from patients for using surgical specimens (study promoter: CHRU Nancy, CPRC2012; sample collection DC2008-459). The patients/participants provided their written informed consent to participate in this study.

## Author Contributions

GG and P-AP designed the study, collected the samples, analyzed the data, and wrote the manuscript. AB collected the samples and the data and analyzed the histological preparations. SC, MB, and ES collected the samples. LM, EL, and PC participated to the elaboration of study design. All authors agree to be accountable for the content of the work.

## Conflict of Interest

The authors declare that the research was conducted in the absence of any commercial or financial relationships that could be construed as a potential conflict of interest.

## Publisher’s Note

All claims expressed in this article are solely those of the authors and do not necessarily represent those of their affiliated organizations, or those of the publisher, the editors and the reviewers. Any product that may be evaluated in this article, or claim that may be made by its manufacturer, is not guaranteed or endorsed by the publisher.
